# Supplementary material for: Conditional chemoconnectomics (cCCTomics) as a strategy for efficient and conditional targeting of chemical transmission
Source: eLife. 2024 Apr 30;12:RP91927. doi: 10.7554/eLife.91927 (PMC11060718; doi:10.7554/eLife.91927)
Supplement: Figure 3—source data 3. [file elife-91927-fig3-data3.zip › Figure 3 source data3 part 1.zip!/Figure 3-Source data 3-part1/1082-9-CRISPResso_Report_zod2ewbs.zip!/CRISPResso_on_1082_11/2a.Nucleotide_percentage_quilt.pdf]

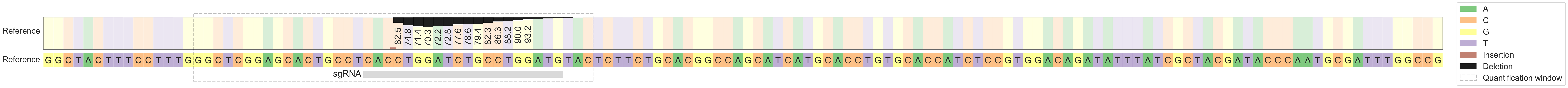
Reference
82.5
74.8
71.4
70.3
72.2
72.8
77.6
78.6
79.4
82.3
86.3
88.2
90.0
93.2
Reference
G G C T A C T T T C C T T T G G G C T C G G A G C A C T G C C T C A C C T G G A T C T G C C T G G A T G T A C T C T T C T G C A C G G C C A G C A T C A T G C A C C T G T G C A C C A T C T C C G T G G A C A G A T A T T T A T C G C T A C G A T A C C C A A T G C G A T T T G G C C G
sgRNA
A
C
G
T
Insertion
Deletion
Quantification window
